# Lateralization of Courtship Traits Impacts Pentatomid Male Mating Success—Evidence from Field Observations

**DOI:** 10.3390/insects13020172

**Published:** 2022-02-05

**Authors:** Donato Romano, Giovanni Benelli, Cesare Stefanini

**Affiliations:** 1 The BioRobotics Institute, Sant’Anna School of Advanced Studies, Viale Rinaldo Piaggio 34, 56025 Pontedera, Italy; cesare.stefanini@santannapisa.it; 2 Department of Excellence in Robotics and AI, Sant’Anna School of Advanced Studies, 56127 Pisa, Italy; 3 Department of Agriculture, Food and Environment, University of Pisa, Via del Borghetto 80, 56124 Pisa, Italy; giovanni.benelli@unipi.it

**Keywords:** courtship behavior, Hemiptera, lateralization, mating behavior, Pentatomidae, reproductive behavior

## Abstract

**Simple Summary:**

Although a growing number of studies have reported asymmetries of brain and behavior in various insect orders, detailed information on lateralization in the courtship and mating behavior of insects in the wild is scarce. In this research, we studied the courtship and mating behavior of the neem bug, *Halys dentatus*, in the field, quantifying lateralized behavioral displays, and assessing their impact on male mating success. A population-level lateralization in males approaching females was found. Furthermore, the male mating success was affected by lateralization; right-biased males achieved higher mating success rates. Overall, our results add useful knowledge on the reproductive behavior of *H. dentatus* in the field, with potential applications for identifying useful benchmarks to monitor the quality of individuals mass-reared for pest control purposes over time. This study furtherly highlights the role of lateralized traits in determining male mating success in insects.

**Abstract:**

Lateralization has been documented in many insect species, but limited information on courtship and mating lateralization in wild conditions is available. We conducted field investigation on the courtship and mating behavior of the neem bug, *Halys dentatus*, a polyphagous insect mainly infesting *Azadirachta indica*, with particular attention to lateralization of mating displays. We investigated the presence of population-level behavioral asymmetries during *H. dentatus* sexual interactions and their influence on male mating success. Two lateralized traits were found: left or right-biased male approaches to the female and left or right-biased male turning displays. Males approaching females from their left side were mainly right-biased in the 180° turning display, and males that approached females from their right side were mainly left-biased. Right-biased males by turning 180° to carry out end-to-end genital contact, performed a lower number of copulation attempts, thus starting copula earlier than left-biased males. Mating success was higher when males approached the left side of females during sexual interactions. A higher number of successful mating interactions was observed in right-biased males when turning 180°. Our results add useful knowledge on the reproductive behavior of *H. dentatus* in the field, with potential applications for identifying useful benchmarks to monitor the quality of individuals mass-reared for pest control purposes over time.

## 1. Introduction

Lateralization (i.e., left–right asymmetries of brain and behavior) is a fascinating principle of the brain organization. It can contribute to improve brain efficiency in cognitive tasks involving both hemispheres, processing several streams of information concurrently [1,2]. Most research focus on lateralized traits in vertebrate animals [3,4,5,6,7,8,9]. However, an increasing number of studies are shedding light on individual- and population-level asymmetries in the brain and behavior of a growing number of insect species [1,2,10,11,12,13,14,15,16]. The lateralization of courtship and mating behavior has been studied in several insect species, including a tephritid fly [17], hymenopteran parasitoids [18,19], stored-product beetles [20,21,22,23], mosquitoes [24], and a calliphorid fly [25]. However, strictly limited information is still available about lateralization of courtship and mating behavior in insect species belonging to the order Hemiptera [10].

The neem bug, *Halys dentatus* (Fabricius) (Hemiptera: Pentatomidae), is a polyphagous insect pest with a marked feeding preference for the neem tree, *Azadirachta indica* (Juss) (Sapindales: Meliaceae), therefore representing a serious threat for this plant species [26]. *Azadirachta indica* is an important plant exploited as source of plant-borne compounds that are effective as green insecticides [27,28,29,30].

The importance of Pentatomidae, one of the largest heteropteran families including more than 4120 described species [31,32], is recognized worldwide. Recently, due to the threat posed by the invasion of some stink bug species, there is an increasing demand to identify sustainable and effective pest management strategies against these insects [33,34,35]. Despite the importance of *H. dentatus* as a pest of *A. indica* trees, little research has been done to investigate the systematics, biology, and behavioral ecology of this bug species [26,36,37]. To the best of our knowledge, little information is available about the mating strategies and the reproductive ethology of the neem bug. 

Previous studies on other pentatomid species have highlighted a notable variety and complexity of their courtship and mating behaviors [38,39,40]. Understanding the behavioral ecology of an insect pest species can be highly beneficial to achieve insights about basic knowledge on courtship and mating, paving the way to innovative strategies in Integrated Pest Management (IPM) [25,41]. Furthermore, no evidence about lateralization is available for the Pentatomidae family, while population-level lateralization of the turning behavior has been reported in the giant water bug *Belostoma flumineum* Say (Heteroptera: Belostomatidae) [10].

Herein, field observations were carried out to analyze and quantify the *H. dentatus* courtship and mating behavior, analyzing the impact of selected behavioral displays on male mating success. While most laterality research have been carried out in laboratory, studying animal behavior in the field is highly recommended to reach realistic conclusions, as pointed out also in entomological research [42]. In the present field study, the magnitude of selected lateralized behaviors was investigated during male–female sexual interactions, to increase our basic knowledge on the *H. dentatus* behavioral ecology, and to achieve insights on the role of lateralized traits among pentatomid bugs.

## 2. Materials and Methods

### 2.1. Field Observations

Field observations were carried out in Delma Park, Abu Dhabi, UAE (24°28′23″ N 54°23′24″ E) from 11:30 to 20:30 h in April and May 2018. *Halys dentatus* courtship and mating behavior were observed in close proximity of plants of *A. indica*, *Acacia nilotica* (L.) Willd. ex Delile, and *Mangifera indica* L., highly infested by this insect. To account for daily variability, behavioral observations were carried out over several days.

*Halys dentatus* subjects were identified according to Chopra [26]. Once a female *H. dentatus* was located, it was focally tracked by an observer for 40 min (or until the end of the sexual interaction, if occurred) [43], to investigate the courtship and mating behavior of *H. dentatus,* as well as if any lateral bias emerged during sexual interactions. The inter-distance between the observer and the focal insects was of ~1 m. The observer was dressed in brown/green clothes (similar to the main pigmentation of the surrounding environment) and settled in such a way as not to shade the insects to minimize his impact on *H. dentatus* behavior. After each mating interaction pentatomids were marked with a small dot of nontoxic color paint (Polycolor, Maimeri, Italy) on the thorax, to avoid repeated observations of the same individuals. In detail, we observed: (i) the time spent by the male performing antennal tapping on the female; (ii) the time spent by the male performing foreleg palpations on the female; (iii) the duration of male’s antennal contact with the backside of the female (i.e., the time spent by the male touching with its antennae the distal part of the female’s abdomen); (iv) the number of the male copulation attempts; (v) the duration of copula (i.e., from the insertion of the male’s aedeagus into the female’s genital chamber until genital disengagement); (vi the male mating success (i.e., if the copula was successful or the female avoided genital contact).

Furthermore, the female’s side approached by the male to move towards the backside of the female (occurring after the antennal tapping and the foreleg palpation displays), as well as the side chosen by the male to turn 180° to allow end-to-end genital linkage and attempt the copula were noted to evaluate the role of lateralized behaviors during *H. dentatus* mating. 

Bugs that were not involved in any courtship and mating interaction were not considered for data analysis. Insects in constrained places were discarded for laterality observations, as well as sexual interactions disturbed by additional individuals [41]. Overall, a total of 29 mating pairs were considered for data analysis.

### 2.2. Statistical Analysis

The impact of lateralization on differences in the mean duration and/or number of courtship and mating acts were analyzed by JMP 16 (SAS Institute, Cary, NC, USA) using nonparametric statistics (*p* < 0.05) as data distribution was not normal (Shapiro–Wilk test, *p* < 0.05), nor homoscedastic (Levene’s test, *p* < 0.05). Laterality differences between the numbers of males approaching the left or right side of the female, as well as the number of males turning 180° to the left or to the right to attempt the copula during courtship interactions were analyzed using a *χ*^2^ test with Yates’ correction (*p* < 0.05 [44]).

## 3. Results

**Figure 1:**
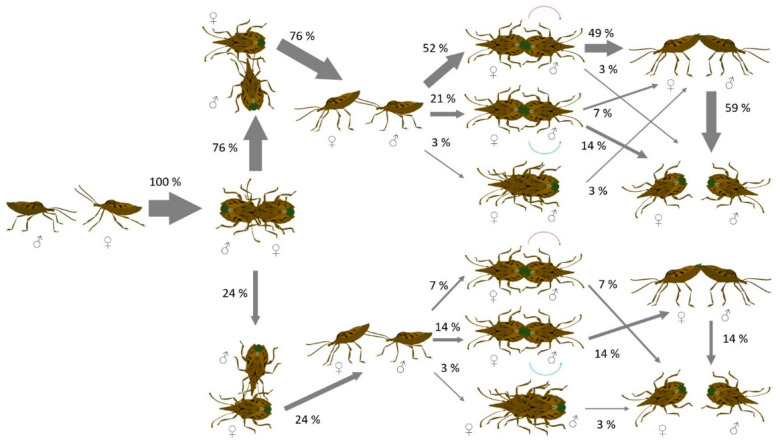
Ethogram depicting the courtship and mating sequence of the pentatomid *Halys dentatus*. The proportion of bugs displaying each behavior is indicated by the thickness of each arrow (n = 29 field-observed mating pairs).

The courtship and mating behavior of *H. dentatus* is described and quantified in Figure 1. When a male intercepts a female, he starts to perform antennal tapping on the surface of her body, as well as to palpate her with its forelegs while moving to the tip of the female abdomen. Subsequently, the male touches with its antennae the distal part of the female’s abdomen that raises her abdomen if receptive. Then, the male rotates his body forming a 180° angle with the female to attempt end-to-end genital contact and to initiate copula. 

**Figure 2:**
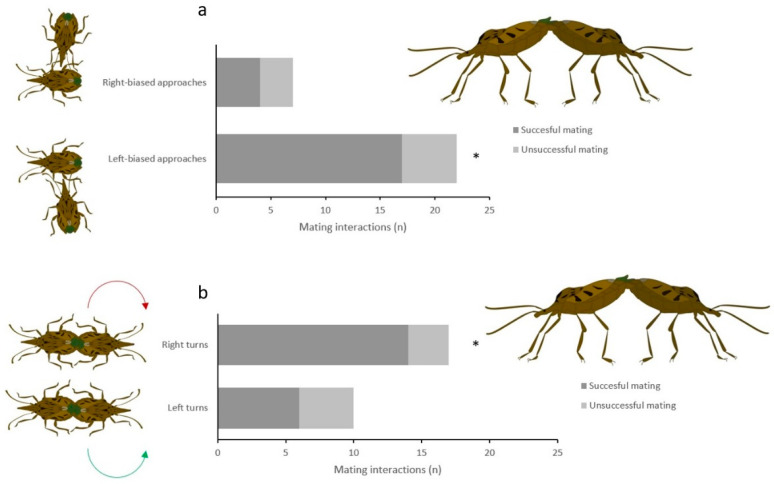
Mating success of *Halys dentatus* males showing (**a**) left or right-biased approaches to the female, and (**b**) left or right-biased turning displays; asterisks indicate a significant difference between left and right-biased acts (*χ*^2^ test with Yates’ correction, *p* < 0.05).

Mating success was higher when *H. dentatus* males approached the females from the left side during sexual interactions (*χ*^2^ = 5.500; *d.f.* = 1; *p* < 0.001), while approaches from the right side did not affect significantly mating success (*χ*^2^ = 0.000 *d.f.* = 1; *p* = 1.000) (Figure 2a); the preferential turning on the right to attempt copula led to higher number of successful males in mating (*χ*^2^ = 5.880; *d.f.* = 1; *p* < 0.001), compared to left-biased turning males (*χ*^2^ = 0.100; *d.f.* = 1; *p* = 0.317) (Figure 2b).

The male mating success was not affected by the duration of the male’s antennal tapping (*χ*^2^ = 1.651; *d.f.* = 1; *p* = 0.198), the duration of the male’s foreleg palpation (*χ*^2^ = 0.828; *d.f.* = 1; *p* = 0.362), the duration of male’s antennal contact with the rear of the female (*χ*^2^ = 0.001; *d.f.* = 1; *p* = 0.980), as well as the number of the male copulation attempts (*χ*^2^ = 3.504; *d.f.* = 1; *p* = 0.061).

No differences in the duration of the male’s antennal tapping (*χ*^2^ = 0.170; *d.f.* = 1; *p* = 0.679), the duration of the male’s foreleg palpation (*χ*^2^ = 0.016; *d.f.* = 1; *p* = 0.897), the duration of male’s antennal contact with the rear of the female (*χ*^2^ = 1.003; *d.f.* = 1; *p* = 0.316), the number of the males’ copulation attempts (*χ*^2^ = 3.527; *d.f.* = 1; *p* = 0.060), as well as the duration of copula (*χ*^2^ = 0.484; *d.f.* = 1; *p* = 0.486), were recorded between left- or right-biased male directional approaches toward females (Table 1). 

The side chosen by the male to turn 180° and attempt the copula did not affect the duration of the male’s antennal tapping (*χ*^2^ = 1.147; *d.f.* = 2; *p* = 0.563), the duration of the male’s foreleg palpation (*χ*^2^ = 0.438; *d.f.* = 2; *p* = 0.803), the duration of male’s antennal contact with the backside of the female (*χ*^2^ = 5.128; *d.f.* = 2; *p* = 0.077), as well as the copula duration (*χ*^2^ = 3.969; *d.f.* = 2; *p* = 0.137).

However, the number of the male copulation attempts was significantly affected by the side chosen by the male to turn 180° and attempt copulation (*χ*^2^ = 16.017; *d.f.* = 2; *p* = 0.0003) (Table 2). Males that turned 180° from their right side performed significantly less copulation attempts to insert their aedeagus into the female’s genital chamber, if compared to left-biased turning males, which started copula earlier.

## 4. Discussion

Insights on the reproductive behavior of insect pests of economic importance are valuable to predict the spatial and temporal population dynamics of these species, allowing adequate modelling paths [45,46], as well as to design effective and sustainable pest management strategies. The recent spreading of pentatomids and their polyphagia are threating agriculture and ecosystems worldwide [33,34,35]. Herein, we provided data from observations in the wild on the courtship and mating behavior of *H. dentatus*, including male antennal tapping on the female body, male foreleg palpation of the tip of the female abdomen, male antennal tapping on the female abdomen, male 180° turning behavior with end-to-end genital contact with the female, followed by the copula. Other pentatomid species which carry out end-to-end copulation have been reported to perform similar courtship sequences [47], such as male antennal tapping on the female, the male attempt to tease the ventral part of the female’s abdomen with its head, as well as the lifting of the abdomen by receptive females [38,47]. The courtship behavior performed by the male seems to be aimed at inducing a proper posture of the female to make the aedeagus insertion easier [38]. 

Interestingly, two behavioral displays of *H. dentatus* were found lateralized at the population level. Most of males moved to the tip of the female abdomen performing antennal tapping and palpating with their forelegs on her left side of the body. Furthermore, males rotated preferentially clockwise (right turns), to form a 180° angle with the female body for the end-to-end genital linkage. Males that approached females from their left side were mainly right-biased in the 180° turning, and males that approached females from their right side were mainly left-biased in the 180° turning. Both these lateralized traits did not affect the main behavioral parameters characterizing *H. dentatus* courtship and mating displays. However, we observed a higher number of copulation attempts performed by males that turned 180° from their left side to form a 180° angle with the female body (Table 2). Likely, right-biased males in turning 180° have a better orientation and make fewer attempts to succeed in genital linkage. 

Notably, the mating success was significantly higher in males that approached the left side of the females, as well as in males that preferentially turned on their right. To the best of our knowledge, this study reporting the lateralized courtship and mating behavior of *H. dentatus*, is the first evidence of population-level lateralized mating traits in the Hemiptera order, where only a motor bias has been reported till now [10]. 

A large body of literature pointed out that population-level lateralized traits are widespread among both social [48,49,50] and solitary insect species. Concerning the latter, a growing number of recent studies are reporting lateralized traits of the courtship and mating in insects, including earwigs (e.g., *Euborellia plebeja* Dohrn, *Labidura riparia* (Pallas)), *Nala lividipes* (Dufour), and *Nala nepalensis* (Burr) [51,52,53], the tephritid fly *Bactrocera oleae* (Rossi) [17], encyrtid parasitoids, *Leptomastidea abnormis* (Girault) and *Anagyrus vladimiri* Triapitsyn [18,19], mosquitoes, *Aedes albopictus* (Skuse) and *Culex pipiens* L. [14,24], as well as the green bottle fly *Lucilia sericata* (Meigen) [41]. In addition, population-level lateralization of mating traits has been found in key stored-product pests, such as the rice weevil, *Sitophilus oryzae* L. [20], the confused flour beetle, *Tribolium confusum* Jaqueline du Val [20,22] the khapra beetle, *Trogoderma granarium* Everts [21], the rust-red flour beetle, *Tribolium castaneum* (Herbst) [23], the larger grain borer *Prostephanus truncatus* (Horn) [54].

According to theoretical models, population-level lateralization is more likely to evolve in social species [55,56]. However, the intense interactions of solitary individuals with their conspecifics (e.g., multiple fighting/mating events), as well as with other species individuals (e.g., predator-prey interactions and host-parasite interactions), may contribute to explain the widespread presence of population-level lateralization in solitary and gregarious species [2,57]. Indeed, what the theory predicts is not that social species need population-level lateralization, but rather that lateralization emerges as an Evolutionary Stable Strategy (ESS) at individual-level or population-level. So, individual-level and population-level lateralization can produce the stability (i.e., an ESS) depending on the context [58]. The fact that different orders of insects exhibit lateralized courtship and mating behaviors suggests that this feature plays an important role as an ESS in the reproductive behavior of the above-mentioned insect species [55]. Most of these research works have been conducted in laboratory conditions. However, it has been argued that investigations in the wild will help to understand the relevance of brain lateralization as a plastic adaptation to ecological demands [59]. Our study provides findings of population-level lateralized courtship and mating in *H. dentatus* in the wild, evidencing an interesting homology with several vertebrates that display lateral biases in their environment, in entirely unconstrained conditions [59,60,61,62,63]. This study, reporting asymmetries in the behavior of insects in their natural environment supports the hypothesis that lateralization in the wild is ubiquitous and need more attention by behavioral biologists.

## 5. Conclusions

Overall, the present research represents a rare report of evidence in the wild of population-level lateralized courtship and mating traits in solitary insects. Of note, it adds basic knowledge to the courtship and mating behavior of invasive pentatomids. In addition, detailed knowledge on the reproductive behavior of *H. dentatus* in the field is of relevance for potential applications aimed to identify behavioral traits to be used as benchmarks to monitor the quality of individuals mass-reared for pest control purposes over time.

Unfortunately, no earlier efforts have been done to assess the presence and functional role of lateralized traits in the Pentatomidae family. Further research efforts are needed. The quantification of mating displays in the field carried out in this study will allow future comparisons with other bug species, to evaluate the possible impact of lateralization on the mating success of pentatomids, thus on their population dynamics, and spread.

## Figures and Tables

**Table 1:** Behavioral displays of *Halys dentatus* males showing side-biased approaches towards the females. values are means followed by standard errors (SE); within each row, similar letters indicate not significant differences between side-biased parameters (Wilcoxon test, *p* < 0.05).

Behavioral Display	Left-Biased Approaches	Right-Biased Approaches	Tested Bugs (n., Left + Right-Biased Bugs)
antennal tapping duration (s)	20.72 ± 1.01 a	21.42 ± 1.58 a	22 + 7 = 29
foreleg palpation duration (s)	15.90 ± 1.05 a	16 ± 2.60 a	22 + 7 = 29
duration of male’s antennal contact with the backside of the female (s)	22.77 ± 0.96 a	21 ± 1.92 a	22 + 7 = 29
copulation attempts (n)	9.77 ± 1.46 a	13.85 ± 2.12 a	22 + 7 = 29
copula duration (min)	76.63 ± 9.65 a	56 ± 20.03 a	17 + 4 = 21

**Table 2:** Behavioral displays of *Halys dentatus* showing lateralized turning behavior. Values are means followed by standard errors (SE); within each row, different letters indicate significant differences among side-biased parameters (Kruskal–Wallis test, *p* < 0.05).

Behavioral Display	Turning 180° Left	Turning 180° Right	Backside Mounting	Tested Bugs (n., Left + Right-Biased + Back Mouting Bugs)
antennal tapping duration (s)	19.9 ± 1.38 a	21.70 ± 1.15 a	19 + 3 a	17 + 10 + 2 = 29
foreleg palpation duration (s)	15.3 ± 2.02 a	16.05 ± 1.11 a	18 ± 6 a	17 + 10 + 2 = 29
duration of male’s antennal contact with the backside of the female (s)	20.1 ± 0.75 a	23.88 ± 1.10 a	20.5 ± 4.5 a	17 + 10 + 2 = 29
copulation attempts (n)	16.8 ± 2.06 a	6.64 ± 0.77 b	15.5 ± 3.5 a	17 + 10 + 2 = 29
copula duration (min)	55.3 ± 15.34 a	84.58 ± 10.52 a	43.5 ± 43.5 a	14 + 6 + 1 = 21

## Data Availability

Data are available on request.
